# Intensity dependency of peripheral nerve stimulation in spinal LTP induced by paired associative corticospinal-motoneuronal stimulation (PCMS)

**DOI:** 10.1371/journal.pone.0259931

**Published:** 2021-11-18

**Authors:** Akira Yamashita, Takenobu Murakami, Noriaki Hattori, Ichiro Miyai, Yoshikazu Ugawa

**Affiliations:** 1 Neurorehabilitation Research Institute, Morinomiya Hospital, Osaka, Japan; 2 Department of Rehabilitation, Ohara General Hospital, Fukushima, Japan; 3 Faculty of Medicine, Department of Neurology, Fukushima Medical University, Fukushima, Japan; 4 Faculty of Medicine, Division of Neurology, Department of Brain and Neurosciences, Tottori University, Tottori, Japan; 5 Faculty of Medicine, Department of Human Neurophysiology, Fukushima Medical University, Fukushima, Japan; Universita degli Studi di Trento, ITALY

## Abstract

Paired associative corticospinal-motoneuronal stimulation (PCMS) induces plasticity at synapses between corticospinal tracts (CSTs) and spinal motoneurons (SMs). We investigated the effects of peripheral nerve electrical stimulation (PNS) intensity on PCMS-induced plasticity. PCMS consisted of 180 paired stimuli of transcranial magnetic stimulation (TMS) over the left primary motor cortex with PNS on the right ulnar nerve at the wrist. We compared effects induced by different PNS intensities: supramaximal, twice and three times sensory threshold intensities. For evaluating efficacy of the synapse between CSTs and SMs, single-pulse TMS was delivered at cervicomedullary junction level, and cervicomedullary motor-evoked potentials (CMEPs) were recorded from the right first-dorsal interosseous muscle before and after PCMS. PCMS with the supramaximal PNS intensity increased CMEP amplitude. The facilitatory effect of PCMS with the supramaximal PNS was larger than those of PCMS with weaker PNS intensities. Sham TMS with the supramaximal PNS showed no CMEP changes after the intervention. PNS intensity of PCMS influences the magnitude of synaptic plasticity induction between the CSTs and SMs at the spinal level, and the supramaximal intensity is the best for induction of long-term potentiation-like effects. The PNS intensity may influence the number of activated SMs by axonal backpropagating pulses with PNS which must overlap with descending volleys induced by TMS.

## Introduction

Paired-associative stimulation (PAS) is a popular noninvasive brain stimulation protocol for inducing synaptic plasticity in humans. By combining transcranial magnetic stimulation (TMS) over the primary motor cortex (M1) with peripheral nerve electrical stimulation (PNS), PAS can induce long-term modulation of sensorimotor cortical excitability [1–4]. The direction and magnitude of the induced plasticity are dependent on the arrival timings of inputs at the sensorimotor cortices. Long-term potentiation (LTP)-like effect is induced either by simultaneous pre- and postsynaptic activation, or by presynaptic depolarization preceding postsynaptic depolarization within a specific time window [2–4].

Several studies have focused on the induction of plasticity at the synapses between the corticospinal tracts (CSTs) and spinal motoneurons (SMs) in the spinal cord [5–10]. This protocol is named the paired (associative) corticospinal-motoneuronal stimulation (PCMS), and descending volleys by TMS over M1 are associated with antidromic inputs from PNS at the synapses between the CSTs and SMs. LTP-like effects by PCMS improved motor function in patients with spinal cord injuries [5,11].

Previous PCMS studies used supramaximal intensity for PNS. However, an issue of stimulus intensity of peripheral inputs has not been studied. The PNS at supramaximal intensity must elicit backpropagation in all the motor neurons. In contrast, PNS at weaker intensities must elicit it in a part of them. TMS must activate a part of SMs and not all of them. If PNS is set at the supramaximal intensity, two synaptic association induced by PCMS should occur in most of the SMs activated by TMS. In contrast, when using weaker PNS intensities, two synaptic association must occur in only a part of SMs activated by TMS or no association may occur in any SMs. We hypothesized that stimulus intensity of PNS is critical for PCMS-induced synaptic plasticity; the supramaximal intensity is the best for the spinal LTP induction. To tackle this issue, we studied the influences of PNS intensity on the LTP-like effects induced by PCMS. We showed that the magnitude of plasticity induction is dependent on the PNS intensity.

## Methods

### Subjects

A total of 19 right-handed healthy volunteers (five females; mean age ± SD, 26.8 ± 7.2 years) participated in the study. Handedness was assessed by the Edinburgh Handedness Inventory [12], and the mean laterality score was 85.8 ± 15.0%. No volunteers took any medication on a regular basis, nor had any neurological or psychiatric diseases [13]. All participants gave written informed consent to participate in this study. This study was approved by the Ethics Committee of Fukushima Medical University (Approval No. 2657) and conformed to the latest version of the Declaration of Helsinki. Fourteen subjects participated in multiple experiments (3 subjects in all Experiments; 2 subjects in Preliminary experiment, Experiment 2, 3, and 5; 5 subjects in Experiment 2, 3, and 5; 4 subjects in Experiment 1 and 4). Five subjects joined in a single experiment (4 subjects in Experiment 1; a subject in Experiment 5).

### Electromyogram recordings

The subjects sat on a comfortable armchair. An electromyogram (EMG) was recorded from the right first-dorsal interosseous muscle (FDI) using surface Ag/AgCl electrodes placed on the center of muscle (recording electrode) and the metacarpophalangeal joint of the index finger (reference electrode). Responses were amplified and bandpass filtered (10 Hz– 3 kHz, Multi Amplifier 1000, DIGITEX LAB Co. Ltd., Japan). Signals were digitized at 5 kHz and data were stored in a computer for later offline analyses (MultiStim tracer; Medical Try System, Japan).

### F-wave recordings

PNS was delivered to the right ulnar nerve at the wrist using an EMG machine (MEB-2200; Nihon Koden, Japan). To elicit the compound muscle action potentials (CMAPs), electric stimuli of rectangular pulses (pulse width 0.2 ms) were delivered using Ag electrodes placed on the skin over the right ulnar nerve 4 cm proximal from the wrist. Supramaximal shocks, adjusted up to the value of 20% higher than the stimulus eliciting maximum CMAP, were delivered at 0.5 Hz to record F-waves.

### Transcranial magnetic stimulation (TMS)

TMS was delivered by a Magstim 200 stimulator (Magstim Co. Ltd, Whitland, Dyfed, UK) connected to a figure-of-eight coil (outer winding diameters, 70 mm). The current waveform was monophasic. The stimulation coil was held tangentially on the scalp at an angle of 45° to the mid-sagittal plane with the handle pointing laterally and posteriorly. The center of the coil junction was placed over the M1 hand area of the left hemisphere. The motor hot spot was determined as the site where TMS consistently elicited the largest MEPs from the right FDI. At the hot spot, we determined the stimulus intensity to elicit MEPs of, on average, 1 mV peak-to-peak amplitude. Then, we determined the resting motor threshold (RMT), the lowest intensity that elicited a response of at least 50 μV in the relaxed FDI in 5 of 10 consecutive trials [14]. The stimulus intensity was adjusted in steps of 1% of the maximum stimulator output. MEP-latency was also measured during a brief contraction of the right FDI muscle.

### Magnetic stimulation at the cervicomedullary junction

We performed single-pulse cervicomedullary junction stimulation (CMS) as reported previously [15]. CMS was applied by a Magstim 200 stimulator connected with a double cone coil (outer winding diameter 110 mm). The coil was placed over the inion, and the coil current flowed downward at the junction region of the coil so that induced current flowed upward in the brain. Cervicomedullary MEP (CMEP) was recorded from the right FDI muscle. The stimulus intensity of CMS was adjusted to elicit a CMEP of 1.0 mV peak-to-peak amplitude during brief contraction of the right FDI.

### Paired (associative) corticospinal-motoneuronal stimulation (PCMS)

PCMS consisted of a combination of a single PNS and a single-pulse TMS. TMS over the left M1 hand area (120% RMT) paired with PNS on the right ulnar nerve at the wrist (supramaximal intensity or other intensities shown later) was repeated 180 times at 0.2 Hz. The definition of the interstimulus interval (ISI) between TMS and PNS followed that by Shulga et al. [16]. We subtracted MEP latency from F-wave latency, and the subtracted value was used to adjust the timing of TMS and PNS. When TMS is given at the subtracted value (ms) later than the PNS, the descending volley by TMS and antidromic volley from the PNS must arrive at the motoneurons at the same time. The ISI of 0 ms means that both inputs reach the motoneurons at the same time. For example, in the case of 20 ms of MEP latency and 27ms of F-wave latency, the ISI of 0 ms indicates that TMS is given 7 ms later than the ulnar nerve stimulation (Table 1). We used the MEP-latency in the active condition because this latency must reflect the timing of the first descending volley reaches spinal motoneurons, and the first volley is the biggest in several descending volleys.

**Table 1:** MEP and F-wave latencies, values of latency adjustment.

	MEP latency	F-wave latency	Values of latency adjustment
Experiment 1 (n = 11)	20.3 ± 0.8	27.5 ± 1.7	7.2 ± 1.2
Experiment 2 (n = 10)			
three times sensory threshold	20.2 ± 0.9	28.2 ± 1.0	8.1 ± 1.0
two times sensory threshold	20.2 ± 0.6	27.7 ± 1.5	7.6 ± 1.3
Experiment 3 (n = 10)	20.0 ± 0.8	28.1 ± 1.1	8.1 ± 1.1
Experiment 4 (n = 7)	20.4 ± 0.7	27.5 ± 1.3	7.1 ± 1.2
Experiment 5 (n = 11)	20.0 ± 0.6	28.1 ± 1.6	8.1 ± 1.4
			means ± SD (ms)

### Experimental design

#### Preliminary experiment: Determination of the optimal ISI for PCMS

This study was conducted to determine the optimal ISI for PCMS to induce the LTP-like plasticity at the spinal cord. Five subjects (one female; mean age ± SD, 28.6 ± 9.7 years) participated in this study. Four different ISIs were selected: an ISI of +3, in which the initial TMS volley reached the motoneurons 3 ms before the arrival of antidromic postsynaptic backpropagating inputs, and ISIs of -4 and -1 ms, in which the antidromic volley derived by PNS reached the SMs in the spinal cord 4 and 1 ms before when the initial descending volley evoked by TMS reached the presynaptic terminal. An ISI of 0 ms means when the antidromic and orthodromic volleys arrive at the motoneurons simultaneously. To obtain the baseline CMEP, we recorded 10 CMEPs 3 times every 5 min before the PCMS intervention because the excitability of baseline must not be completely the same for a long time. We used the mean size of all 30 CMEPs as the baseline CMEP-size. To follow up CMEPs, 10 CMEPs were recorded at 10, 20, 30, 40, 50, and 60 min after the intervention, and the mean size of 10 CMEPs was used as the post-CMEP size at each time point. We recorded only 10 CMEPs at one time point after the intervention to exclude the possibility that CMEP size may change in time when recording 30 CMEPs continuously. We used a size ratio of the mean CMEP after intervention to the baseline CMEP as a marker of the effect of PCMS. The average time course of the size ratio was depicted by the time after intervention as the abscissa, and the average size ratio from all subjects as the ordinate.

Because the time after the PCMS intervention not including the baseline time point did not affect the size ratio (shown below), we calculated the mean of size ratios at all times after the intervention as the value representative of the long-term effect induced by PCMS at a certain TMS-PNS interval, known as the integral size ratio (ISR) at a certain TMS-PNS interval.

**Fig 1:**
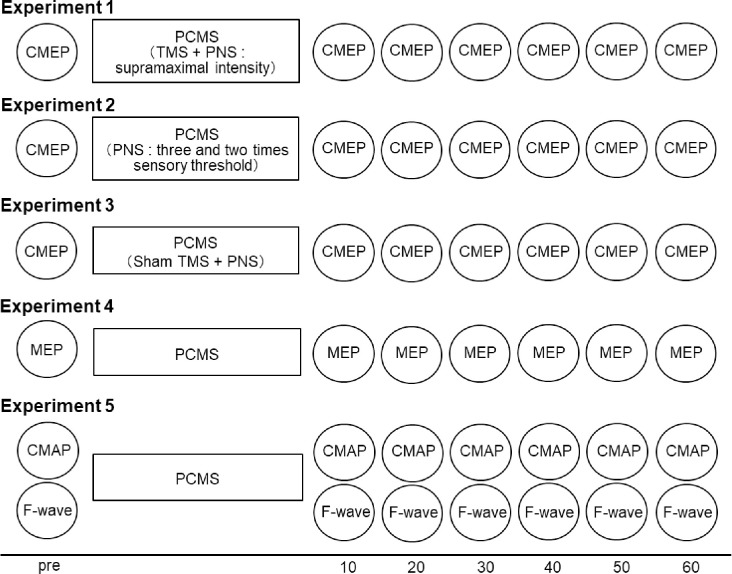
Experimental design.

Experimental design was shown in Fig 1.

#### Experiment 1: Induction of LTP-like synaptic plasticity at the spinal level (Fig 1)

Eleven subjects (four females; mean age ± SD, 26.8 ± 7.2 years) participated in this study. We used the optimal ISI (0 ms) to induce LTP-like synaptic plasticity based on the above preliminary experiment. The experimental procedures were the same as the preliminary experiment.

#### Experiment 2: Effect of PNS intensity on PCMS (Fig 1)

This experiment was conducted to clarify how the intensity of PNS influences synaptic plasticity induction at the spinal cord. Ten subjects (two females; mean age ± SD, 31.9 ± 7.7 years) participated in this experiment. The stimulus intensities of PNS were set at three times sensory threshold inducing a small muscular twitch in the FDI and at two times sensory threshold without any twitches. These results were compared with those of the supramaximal stimulation intensity in Experiment 1.

#### Experiment 3: Sham TMS with real PNS (Fig 1)

This experiment was performed to confirm the necessity of both TMS over M1 and supramaximal PNS for synaptic plasticity induction. Ten subjects (two females; mean age ± SD, 31.9 ± 7.7 years) participated in this study. PNS were delivered to the right ulnar nerve at the wrist. The pulse width was 0.2 ms, and the stimulus intensity was set at supramaximal intensity. The TMS coil was tilted away from the scalp at a 90° angle with one wing of the figure eight touching for sham stimulation [17]. The magnetic stimulator was discharged at the same timings used in the Experiment 1 in each subject. The sham TMS with PNS was repeated 180 times at 0.2 Hz for 15 min. The results of this experiment were compared with those of the LTP of Experiment 1.

#### Experiment 4: Effects of PCMS on MEP to TMS over M1 (Fig 1)

The aim of this experiment was to confirm that cortical MEPs were also modulated similarly to CMEPs by PCMS at the supramaximal PNS intensity. This is because the cortical MEP must reflect a combination of excitability of supraspinal components and that of spinal motoneuronal component, whereas the CMEP must reflect only spinal motoneuronal excitability. To evaluate the effects on MEPs, single-pulse TMS was delivered over M1 before and after the PCMS intervention at the same timings in the Experiment 1. Seven subjects (one female; mean age ± SD, 22.7 ± 0.5 years) participated in this study. Single-pulse TMS was applied to the left M1 hand area and MEPs were recorded from the active right FDI muscle. The intensity of TMS was set to elicit approximately 1 mV MEPs in baseline condition. The time course of MEP recording was the same in the above experiments.

#### Experiment 5: Effects of PCMS on the peripheral nervous system (Fig 1)

This experiment was conducted to investigate effects of PCMS at the supramaximal PNS intensity on the excitability of the peripheral nervous system. Compound muscle action potentials (CMAPs) and F-waves were measured before and after the PCMS intervention at the same timings in the Experiment 1. Eleven subjects (two females; mean age ± SD, 30.8 ± 8.2 years) participated in this experiment. Both responses were measured before and after PCMS.

Subjects participated in Experiment 1–5 in a randomized order. Any successive interventions were separated by at least one week in the same subject.

### Statistical analysis

All statistical analyses were performed by using IBM SPSS Statics version 27 for Windows software.

The baseline CMEP amplitude and stimulus intensity to elicit 1 mV CMEP were compared between different experiments using one-way analysis of variance (ANOVA) or an unpaired *t*-test.

In Preliminary experiment and Experiments 1–3, the CMEP size ratio at each time point was used as the marker of the effect. In Preliminary experiment, a two-way repeated measures ANOVA (rmANOVA) with time not including the baseline time point (post 10–60 min: 6 levels, only those after the intervention) and intervention (ISIs: -4, -1, 0, +3 ms: 4 levels) as the within-subject factor. To compare the effects of PCMS protocols in Experiments 2 and 3 with those in Experiment 1, the CMEP size ratio was evaluated by two-way repeated measures ANOVAs (rmANOVAs) of mixed design with time (post 10–60 min: 6 levels) as the within-subject factor and intervention (Experiment 2: PCMS at supramaximal intensity vs. PCMS at three times sensory threshold vs. PCMS at two times sensory threshold: 3 levels; Experiment 3: PCMS vs. sham TMS-PNS pair: 2 levels) as the between-subject factor. In the case of significant main effects or interactions, *post-hoc* analyses were performed using a one-sample *t*-test in Preliminary experiment, and a paired *t*-test with Bonferroni-correction for multiple comparisons in Experiment 2.

In Experiments 1–5, to compare post-PCMS effects with the baseline, the absolute data of the amplitudes were analyzed using one-way rmANOVA with time (pre, post 10–60 min: 7 levels, including the baseline time point) as the within-subject factor. When a significant main effect was observed, a *post-hoc* paired *t*-test with Bonferroni-correction for multiple comparisons was conducted.

To assure no differences in background EMG activity throughout the experiments, integrated EMG (iEMG, mVmsec) amplitudes were calculated in a window from 10 ms before to the onset of the TMS pulse and analyzed it using one-way rmANOVAs with time (pre, post 10–60 min: 7 levels).

In all tests, a value of *P* < 0.05 was considered statistically significant. Data were expressed as the mean ± standard error of the mean.

## Results

### Preliminary experiment: Determination of the optimal ISI for PCMS

There was no significant difference in the baseline CMEP amplitude among ISIs (*F*_(3,16)_ = 0.002, *P* = 1.0).

**Fig 2:**
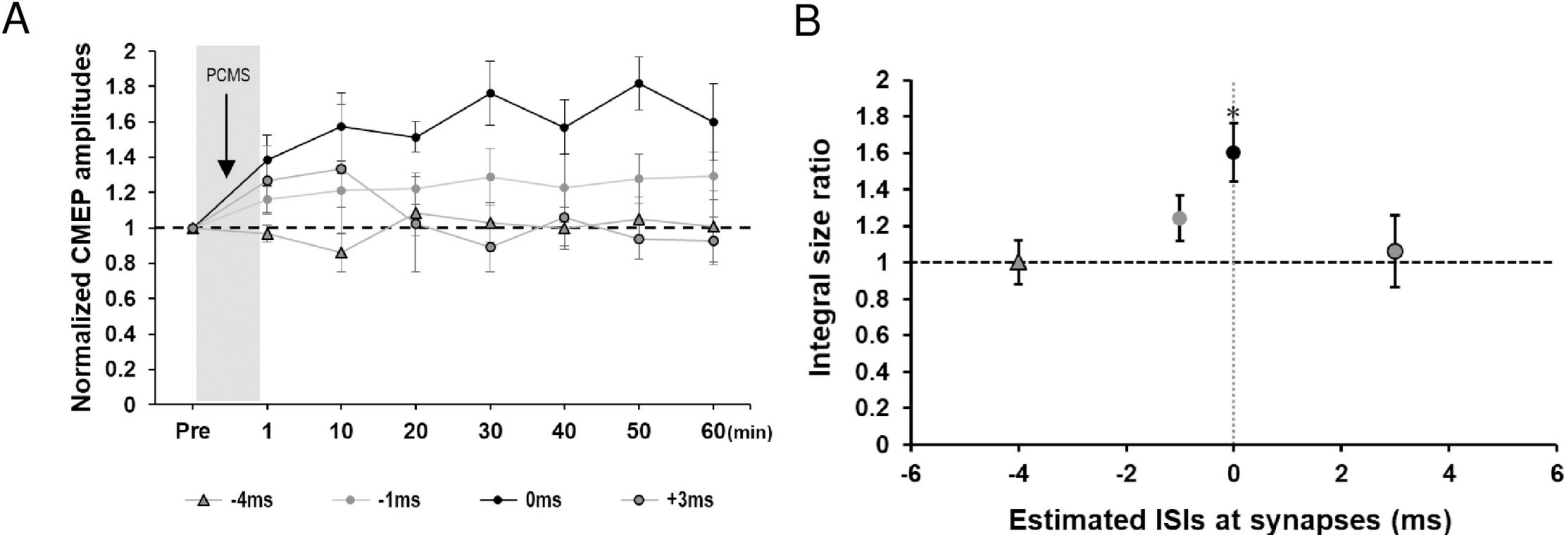
CMEP amplitudes after PCMS in Preliminary experiment. (A) Time courses of the average CMEP size ratio from 10 to 60 min after PCMS using a supramaximal PNS at ISIs of -4, -1, 0, and +3 ms. CMEP amplitude increased after PCMS with an ISI of 0 ms. (B) The integral size ratio (ISR) at various estimated ISIs at motoneurons (TMS-PNS interval). The ISR increased significantly at the ISI of 0 ms. * *P* < 0.05. All data are mean and standard-error values.

Fig 2A shows time courses of the average size ratio for PCMS at ISIs of -4, -1, 0, and +3 ms. A two-way rmANOVA showed a significant main effect of intervention (*F*_(3,12)_ = 5.020, *P* = 0.018) but no significant main effect of time after the intervention not including the baseline time point (*F*_(5,20)_ = 0.205, *P* = 0.956) or interaction (*F*_(15,60)_ = 1.086, *P* = 0.388). The ISR was significantly different among the TMS-PNS intervals. The ISR was significantly greater than 1.0 at the ISI of 0 ms (*P* < 0.05) (Fig 2B).

Hence, the optimal ISI of PCMS was 0 ms for induction of the LTP-like effect. Based on these results, we used ISI of 0 ms in all the following experiments.

### Experiment 1: Induction of LTP-like synaptic plasticity at the spinal level (PCMS using a supramaximal PNS)

**Fig 3:**
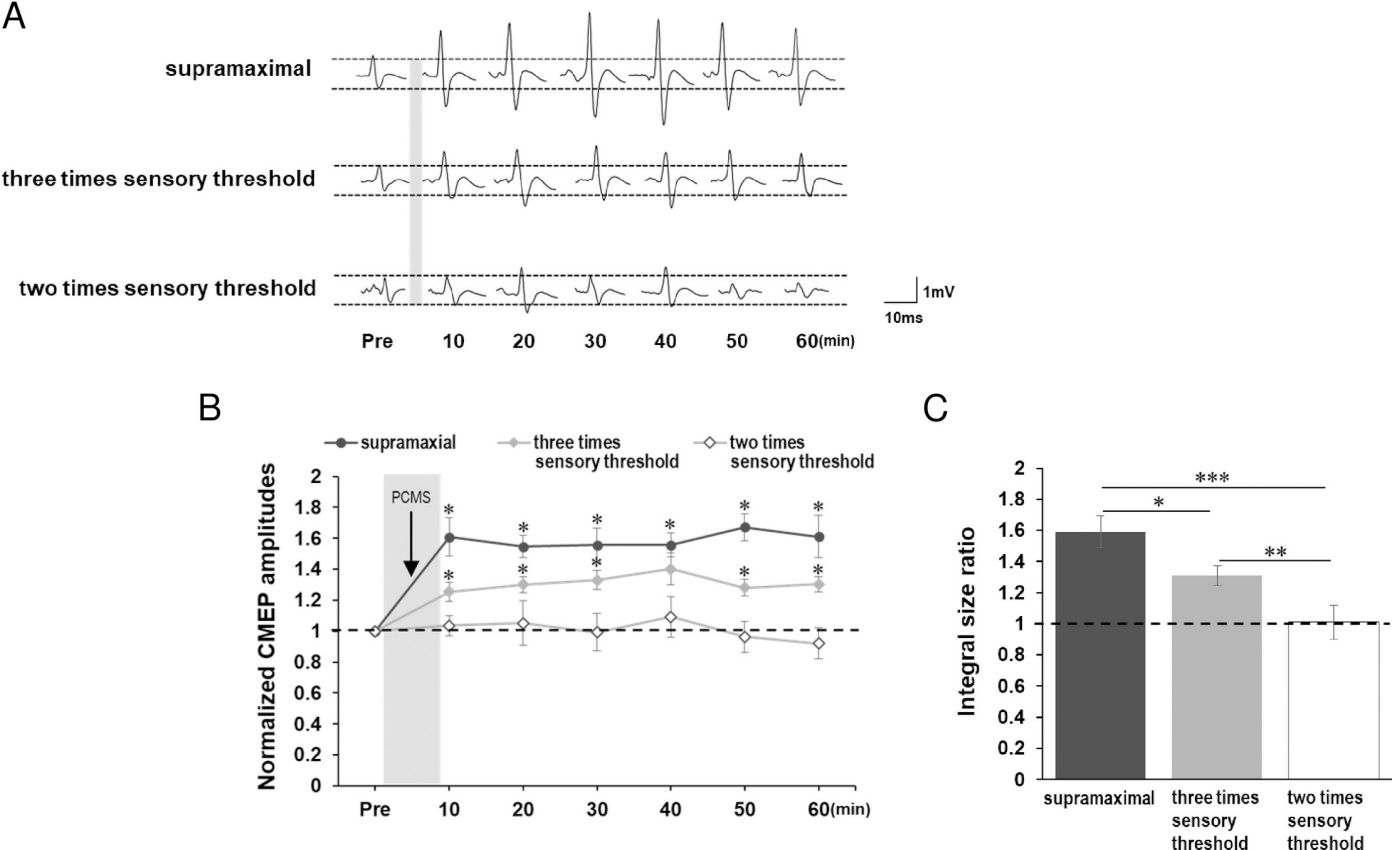
Normalized CMEP amplitudes after PCMS in Experiment 1 and influences of PNS intensity on PCMS effects in Experiment 2. (A) Representative CMEP waveforms before and after the intervention. (B) Time courses of the average CMEP size ratio from 10 to 60 min after PCMS at supramaximal PNS intensity, three times the sensory threshold, and two times the sensory threshold. CMEP increased after PCMS at supramaximal PNS intensity and at three times the sensory threshold, while PCMS at two times the sensory threshold showed no significant changes. (C) The ISR of PCMS. The ISR of PCMS at the supramaximal PNS was the greatest among the protocols. The ISR of PCMS at three times the sensory threshold was larger than that of PCMS at two times the sensory threshold. * *P* < 0.05, ** *P* < 0.01, *** *P* < 0.001. All data are mean and standard-error values.

To compare post-PCMS effects with the baseline, a one-way rmANOVA disclosed the significant main effect of time (all time points including baseline time point) (*F*_(6,60)_ = 7.915, *P* < 0.001). *Post-hoc* tests showed that CMEP amplitudes significantly increased after PCMS (10–60 min, *P* < 0.05) (Fig 3A and 3B).

This result indicated that PCMS using a supramaximal PNS induced LTP-like plasticity at the synapses between CSTs and SMs.

### Experiment 2: Impact of PNS intensity on PCMS effect

The CMEP amplitude at baseline was not significantly different among all PNS intensities (*F*_(2,28)_ = 0.383, *P* = 0.685).

Fig 3A shows representative waveforms of CMEP at all time points and Fig 3B shows time courses of the average size ratio for PCMS using three, two times sensory threshold and the supramaximal PNS. A two-way rmANOVA showed a significant main effect of intervention (*F*_(2,28)_ = 29.230, *P* < 0.001) but no significant main effect of time after the intervention (not including the baseline time point) (*F*_(5,140)_ = 0.293, *P* = 0.916) or interaction (*F*_(10,140)_ = 0.575, *P* = 0.833), suggesting that the ISR was significantly different among the PNS intensities. *Post-hoc* comparisons demonstrated that the ISR of PCMS at the supramaximal PNS intensity was the strongest among the protocols (PCMS at three times sensory threshold, *P* = 0.03; PCMS at two times sensory threshold, *P* < 0.001). The ISR of PCMS at three times sensory threshold was larger than that of PCMS at two times sensory threshold (*P* = 0.002) (Fig 3C).

To compare post-PCMS effects with the baseline, a one-way rmANOVA showed a significant main effect of time in PCMS at three times sensory threshold (*F*_(6,54)_ = 3.870, *P* = 0.003). A *post-hoc* comparison showed that CMEP increased after intervention (10–30, 50–60 min, *P* < 0.05). No significant main effect of time was observed in PCMS at two times sensory threshold (*F*_(6,54)_ = 0.594, *P* = 0.734) (Fig 3B).

This result suggested that PNS intensity had an influence on the ISR in a dose-dependent manner. Namely, the supramaximal PNS may be needed to induce a stable PCMS effect.

### Experiment 3: Sham TMS with real PNS

We compared the effects between PCMS in Experiment 1 and the sham TMS-PNS pair in Experiment 3 using the CMEP size ratios at time points after the intervention not including the baseline data. A two-way rmANOVA revealed a significant main effect of intervention (*F*_(1,19)_ = 39.198, *P* < 0.001) but no significant main effect of time (*F*_(5,95)_ = 0.618, *P* = 0.687) nor interaction (*F*_(5,95)_ = 0.040, *P* = 0.999).

**Fig 4:**
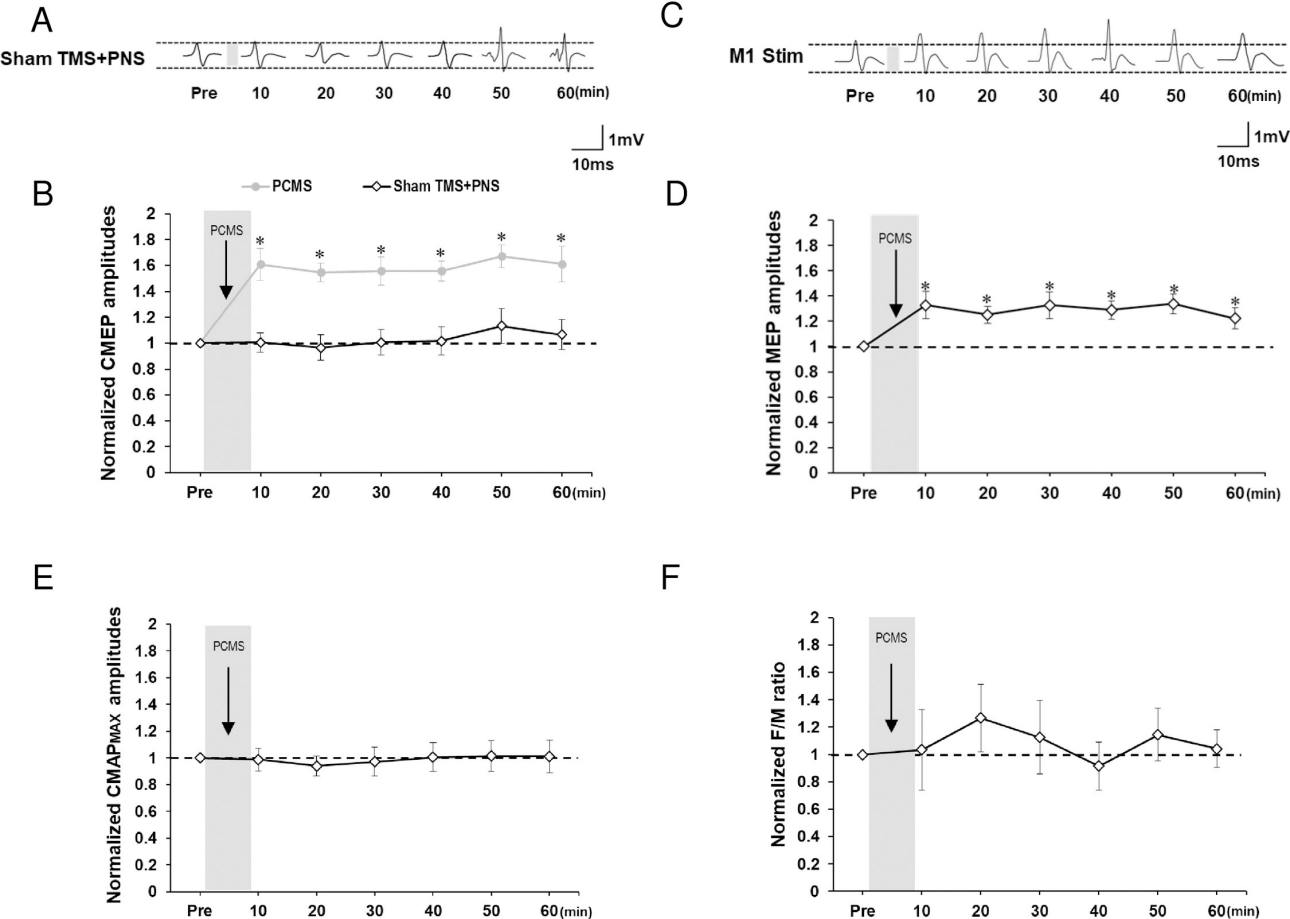
Time courses of PCMS effect in Experiment 3–5. (A) Representative CMEP waveforms before and after sham TMS-PNS pairs. (B) Repeated sham TMS-PNS pairs induced no significant changes in CMEP amplitudes. (C) Representative MEP waveforms before and after PCMS. (D) Cortical MEP amplitudes significantly increased after PCMS compared to the baseline. (E) CMAP amplitudes remained unchanged after PCMS. (F) PCMS did not affect the F/M ratio. * *P* < 0.05. All data are mean and standard-error values.

For the time course of the sham TMS with real PNS, a one-way rmANOVA using the raw CMEP data showed no significant effect of time (*F*_(6,54)_ = 0.239, *P* = 0.962), indicating that the repeated sham TMS-PNS pair had no effect on CMEP sizes (Fig 4A and 4B).

### Experiment 4: Effects of PCMS on MEP to TMS over M1

A one-way rmANOVA showed a significant main effect of time (*F*_(6,36)_ = 2.516, *P* = 0.039). The *post-hoc* analysis showed the increments of the MEP amplitudes after PCMS for 60 min (10–60 min, *P* < 0.05) (Fig 4C and 4D).

### Experiment 5: Effects of PCMS on the peripheral nervous system

One-way rmANOVAs showed no significant effect of time on CMAP (*F*_(6,60)_ = 0.414, *P* = 0.867) or on the F/M ratio (*F*_(6,60)_ = 0.350, *P* = 0.907) (Fig 4E and 4F).

### Background EMG activity

There was no significant effect of time on the background iEMG in Preliminary experiment and Experiments 1–3 (*F* < 1.3, *P* > 0.3, Table 2).

**Table 2:** Results of one-way rmANOVAs in background iEMG.

Experiment	df	Error	*F*	*P*
Preliminary experiment				
ISI of -4 ms	6	24	0.576	0.746
ISI of -1 ms	6	24	0.357	0.899
ISI of 0 ms	6	24	0.427	0.853
ISI of +3 ms	6	24	1.224	0.329
Experiment 1	6	60	1.087	0.381
Experiment 2				
three times sensory threshold	6	54	0.799	0.575
two times sensory threshold	6	54	0.294	0.937
Experiment 3	6	54	0.724	0.632

## Discussion

The present study investigated the synaptic plasticity induction at the synapses between CSTs and SMs at the spinal cord level following the rule of paired associative plasticity. The facilitatory effects of PCMS with the supramaximal PNS was much stronger than those of PCMS with weaker PNS, indicating that PNS intensity has a great influence on the magnitude of induced plasticity.

### Timing of TMS and PNS in PCMS

Previous PCMS studies suggested that the LTP-like plasticity is induced when corticospinal volleys arrive at the corticomotoneuronal synapses 1–2 ms prior to antidromic inputs of postsynaptic motoneurons [5,8,18]. Other studies reported that the LTP-like effects are successfully induced when initial descending volley from TMS and the antidromic inputs by PNS reach spinal motoneurons at the same time [7,11,16]. The present results are consistent with the latter idea. Theoretically, stimulus timing is essential for inducing synaptic plasticity in a rule of the associative plasticity (spike timing-dependent plasticity). Much evidence confirms that the LTP is induced on presynaptic spike preceding the postsynaptic spike within a narrow time window, or on simultaneous presynaptic and postsynaptic depolarization [19]. We speculate that the setting of ISI within 1–2 or 0 ms was not critically different for inducing the LTP-like effects by PCMS from our results.

### Site of action in PCMS

In the present study, PCMS increased CMEP and did not affect CMAP nor F/M ratio. These findings suggest that PCMS should induce plasticity at the synapses between CSTs and SMs but not on the peripheral nervous system, which are consistent with a previous report [5]. Both TMS over M1 and PNS are needed for the effect shown here, because sham TMS with real PNS had no effects on CMEPs in the present results and TMS only showed no MEP changes in the previous study [7]. These results are consistent with previous reports [5,8]. In addition, PCMS increased the MEP amplitudes elicited by single-pulse TMS over the contralateral M1. This result is in line with previous reports [7,16,20], and probably due to spinal LTP and not cortical LTP.

The LTP is induced if a pair of presynaptic and postsynaptic depolarizations is evoked simultaneously or within a narrow time range. Supramaximal stimulus of peripheral nerve directly activates all motor neurons antidromically, and TMS produces burst firing of SMs by descending volleys through several mechanisms [21]. SM bursting and depolarization duration of the SM membrane potential play important roles in determining the timing for the direction of synaptic plasticity induction. LTP may be induced at the time when both effects should be associated in time at the synapses, especially when both inputs arrive at the SM at the same time (the interval of 0 ms).

In the present experimental setting, PNS should evoke motor axonal backfiring, which induces depolarization and action potential of SMs. TMS elicits multiple descending volleys in CSTs which contribute to the bursting of SMs. The first descending volley is the biggest in several volleys and the timing can be estimated by the MEP-latency in the active condition. The best timing for LTP is when both effects arrive at the same time and the strongest association must occur at the latency of MEP in active condition. This speculation is consistent with our present result that ISI of 0 ms is good for LTP.

### PNS intensity and plasticity induction by PCMS

The present study, for the first time, demonstrated that the intensity of PNS has an impact on the magnitude of the induced plasticity. An antidromic impulse induced by PNS arrives at the axon hillock of the SMs. However, the majority of SMs is not always activated even by PNS with supramaximal intensity because of an impedance mismatch at the axon hillock [22]. PCMS can induce strong LTP-like effects at the synapses between CSTs and SMs only when activation of many SMs by PNS at supramaximal intensity overlaps with TMS-induced descending volleys. On the other hand, because PNS at weaker intensity may activate only a small number of SMs antidromically, two synaptic association induced by PCMS may occur in a part of SMs or may not occur in any SMs. Small degree of LTP-like effects induced by PCMS at three times sensory threshold could be explained by that two synaptic association might occur in a part of SMs. No induction of synaptic plasticity by PCMS at two times sensory threshold might explain no synaptic association in the SMs. Whichever the mechanisms for small plasticity induction, our conclusion is still correct that the supramaximal PNS is necessary for inducing the LTP-like effects by PCMS.

### Study limitations

Similar to other non-invasive brain stimulation protocols [23–25], the interindividual variability should be considered in PCMS [26]. The number of subjects was relatively small and the population of subjects was not identical among experiments in the present study. These factors might possibly explain the difference in the aftereffects between the different intensity PCMSs in the present investigation. To make a firm conclusion about this factor, we need future studies of the variability in effects of PCMS in greater number of subjects.

In conclusion, PCMS could successfully increase excitability at the synapses between CSTs and SMs at the spinal level. The magnitude of the induced plasticity is dependent on the PNS intensity, which may reflect the number of the antidromically activated SMs by PNS. Induction of synaptic plasticity by PCMS is associated with improvement of motor functions [5,11], and PCMS will have potentials to develop as a nonpharmacological therapeutic application for patients with spinal cord diseases.
